# Insulin‐Related Disordered Eating Behavior: Clarifying Terminology

**DOI:** 10.1002/eat.24548

**Published:** 2025-09-16

**Authors:** Amy Shelford, Paul E. Jenkins, Kate Harvey

**Affiliations:** ^1^ School of Psychology and Clinical Language Sciences University of Reading, Whiteknights Campus Reading UK

**Keywords:** disordered eating behavior, insulin, T1DE, type 1 diabetes

## Abstract

**Objectives:**

Consistent terminology for disordered eating behaviors relating to insulin manipulation is needed to consolidate and harmonize knowledge across research and practice. The aim of this study was to explore the terminology used to describe insulin‐related disordered eating behaviors in people with type 1 diabetes and suggest consolidated terms to be used consistently going forward.

**Method:**

This study was conducted as part of a larger scoping review and offers analysis of terms used in the literature.

**Results:**

Across 201 primary and secondary sources, 19 different terms were identified, 11 of which related specifically to insulin restriction. Motivations for insulin manipulation were rarely defined.

**Discussion:**

*Insulin‐related disordered eating behavior* is the proposed umbrella term to encompass all facets and directions of insulin manipulation, including *insulin inflation* and insulin restriction. Providing a consistent term for use in this field will consolidate evidence, facilitating a clear research direction and increasing the visibility of evidence to be used in practice recommendations and care pathways.

**Summary**

- Consequences of insulin‐related disordered eating behavior compound those of eating disorders and poor diabetes management.
- Currently, a range of terminology and definitions is used to represent key behaviors, impeding researchers, healthcare professionals, and patients.
- Consolidation of terms used to refer to these behaviors will improve visibility and accessibility of evidence in the field, which will help to guide and improve future research and practice recommendations.

## 1 Background

Disordered eating is suggested to be more common in individuals with type 1 diabetes (T1D) than their non‐diabetic peers (López‐Gil et al. 2023; Niemelä et al. 2024). Insulin manipulation can be considered a disordered eating behavior when additional insulin is taken to “permit” eating, or a dosage is restricted to control weight (Bauman et al. 2018; Goebel‐Fabbri 2008).

T1D is an autoimmune disease resulting in the inability to produce insulin and metabolize glucose, necessitating self‐management through counting carbohydrates and proportionate administration of insulin through multiple daily injections or constant subcutaneous insulin infusion (“insulin pump”). The inherent focus on food and weight in clinic appointments, alongside typical pre‐diagnosis weight loss and subsequent regain with reinsulinization, is suggested to heighten the risk of disordered eating in T1D (Abild et al. 2024). Psychosocial elements of T1D management, such as diabetes distress and the potential for the management regimen to promote negative attitudes towards insulin as “fattening” and food as something to be strictly controlled and compensated for, are implicated as risk factors for developing disordered eating (Harrison et al. 2021; Poos et al. 2023). Insulin manipulation leads to poorer metabolic control and greater risks of complications such as retinopathy, neuropathy, and nephropathy (Bauman et al. 2018; Goebel‐Fabbri 2008; Nip et al. 2019).

Insulin restriction for weight control is inconsistently referred to as diabulimia, insulin omission syndrome, and type 1 diabetes disordered eating (T1DE), among other terms, causing confusion for patients, researchers and healthcare professionals (Brewster et al. 2020). Similarly inconsistent reference to administration of additional insulin has resulted in a lack of awareness as a disordered eating behavior. It is therefore difficult to identify and synthesize reliable information regarding etiology, consequences and recommendations, limiting the extent to which meaningful conclusions can be drawn. The visibility of findings is also reduced, impeding the development of clear research directions and practice recommendations as demonstrated by changes in terminology in other areas (Dorri 2025). For example, changing from “shell shock” to “Post‐Traumatic Stress Disorder” promoted consolidation of research and treatment direction and outputs, and continued revision may reduce stigma and increase access to treatment (Chekroud et al. 2018; Heber et al. 2023). These are important implications for consolidating terms in the area of insulin manipulation. Finally, the inconsistent use of terms has the potential to cause conflict and confusion in people living this experience, if they are unfamiliar or perceived as the “wrong” label (e.g., Allan 2015), which may also impact valid measurement and screening. “Long COVID” is a term originally proposed by patients but requires further development to accurately communicate incidence, subtyping, and pathophysiology (e.g., see Munblit et al. 2022).

A clear, consistent definition of insulin misuse for eating or weight‐related goals can foster clear communication and advancement of knowledge and interdisciplinary communication (Mamarasulova 2024). Moves towards standardizing terminology in the area of insulin manipulation could organize research on this topic and help improve outcomes for those affected (Dorri 2025; Mamarasulova 2024).

Given that the implications of inconsistencies in terminology may include physical and psychological consequences of insulin manipulation, we investigated the terminology used to describe disordered eating behavior related to insulin use. Having identified relevant papers as part of a larger scoping review (in preparation), the terms used in papers were analyzed in this study with a view to summarizing usage across the area and proposing a term that can be used consistently going forward.

## 2 Method

This study was conducted as part of a larger scoping review which aimed to synthesize research on insulin‐related disordered eating behavior (IRDEB) and clarify directions for future research and practice. As it was a review of existing evidence, ethical approval was not required. Methods relating to the aims of this paper are outlined briefly below.

### 2.1 Protocol and Registration

The scoping review was designed and conducted in accordance with Joanna Briggs Institute (Peters et al. 2020) and PRISMA‐ScR guidelines (Page et al. 2021; Peters et al. 2020). Full details of the methods of the wider scoping review are openly available in the pre‐registered protocol (Shelford et al. 2023).

### 2.2 Searches and Eligible Evidence

**FIGURE 1:**
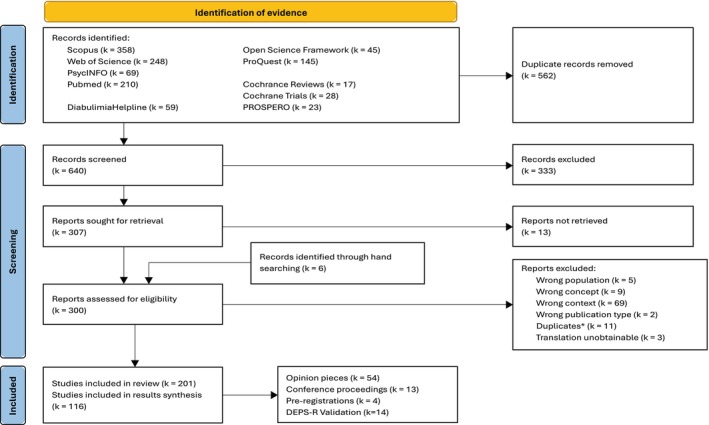
PRISMA flow diagram. *Where duplicates were available in different formats, such as a thesis and a journal article, or conference proceeding and article, the source that offered the most evidence pertaining to the research questions was kept. DEPS‐*R* = Diabetes Eating Problem Survey Revised.

Systematic searches were performed in PsycINFO, PubMed, Scopus, Web of Science, ProQuest, Open Science Framework, Cochrane Library, and PROSPERO, alongside the literature section of charity website *Diabulimia Helpline* for evidence investigating individuals with T1D manipulating insulin, either under‐ or overdosing, for the purpose of controlling weight or calorie consumption. The search strategy (see Supporting Information) used Boolean operators and truncation to cover terms relating to type 1 diabetes AND insulin manipulation for disordered eating NOT suicide. Evidence on type 2 diabetes, alternative motives for insulin manipulation, and news articles, books, and book chapters evidence types were excluded. Search results were 100% double‐screened by two independent reviewers at the title and abstract level, and 10% at the full‐text level (see Figure 1).

### 2.3 Data Charting

Charting involved the extraction of all terms used to refer to the behaviors of interest from the introductions and abstracts of all sources included in the scoping review, including empirical studies, reviews, opinion pieces, unpublished work, conference proceedings, and pre‐registered protocols. Types of insulin manipulation behaviors with disordered eating motivations were categorized and used to derive inclusive and representative terms. This was also supported by related evidence discussing terms and language connotations.

### 2.4 Synthesis of Results

Frequency counts summarize the terms used and contexts of the behaviors of interest. Frequency of terms, semantics, and supporting literature on established connotations and types of insulin manipulation were used to develop terminology suggested for use going forward.

## 3 Results

Terminology referring to IRDEB varied substantially across publications, with 19 different terms identified in this review. Of these terms, two related to the use of insulin above the prescribed dose; 11 terms related to the use of less insulin than a prescribed dose; six terms were not directly attributed to a direction of insulin manipulation. Some sources used multiple terms and phrases, resulting in 319 uses of different terms and phrases across sources (see Table 1).

**TABLE 1:** Frequency count of terms referring to insulin‐related disordered eating behavior across all evidence.

Direction of insulin manipulation	Terms used	Count
Taking less insulin than a prescribed dose	Insulin omission (including variations of “omit”)	112
	Diabulimia	45
	Insulin restriction (including variations of “restrict”)	44
	Insulin reduction	12
	Underdosing (of insulin)	10
	Skipping (insulin/doses)	7
	Decreasing (insulin/doses)	4
	Underusing (insulin/doses)	3
	Withholding insulin	3
	Insulin purging	2
	Limiting insulin (doses)	2
Taking more insulin than a prescribed dose	Overdosing (insulin)	3
	Factitious hypoglycemia	1
Unattributed	Insulin misuse	32
	Insulin manipulation (including variations of “manipulate”)	24
	Eating Disorders in Diabetes Mellitus Type 1 (ED‐DMT1)	4
	Type 1 Diabetes and Disordered Eating (T1DE)	8
	Diabetes mismanagement	2
	Insulin abuse	1

Behaviors were contextualized primarily by insulin (164 cases), by diabetes (16 cases) and, infrequently, as medication or drug use (2 cases). In two instances, the behavior was described as inappropriate within the phrase representing the behavior (e.g., “inappropriate manipulation of insulin”; Bubb and Pontious 1991, 31). Nineteen cases contextualized the behavior as intentional (e.g., “intentional insulin omission”; Pinhas‐Hamiel et al. 2013, 819). A body weight‐related element was attributed as a motivation for the behavior of interest in 36 cases (e.g., “insulin restriction for weight control”; Beam et al. 2021, 1110). In the majority of cases, despite the sources focusing on disordered eating, motivation for the behaviors of interest was not included in the phrases used.

Triangulating these results with types of insulin manipulation behaviors identified and charted from all primary sources, and connotations from areas of medicine and social sciences, terms to be used consistently going forward were devised and are summarized in Table 2.

**TABLE 2:** Summary of proposed recommended framework, terms, definitions and rationale.

Proposed term	Definition	Rationale
Insulin‐related disordered eating behavior (IRDEB)	Umbrella term to refer to all disordered eating behaviors involving the manipulation of insulin	Avoids connotations with diagnosable eating disorders. Inclusive of: Manipulation of insulins with different activity profiles Different mechanisms of manipulation Various motivations that are related to disordered eating
Active IRDEB	Deliberate engagement with manipulating insulin doses or diabetes care practices with the intention of achieving hypo‐ or hyperglycemia with disordered eating motivations	Avoids blame connotations with “intention” Relates to health behavior
Passive IRDEB	Disengagement with appropriate diabetes‐related care practices that would highlight or remedy blood glucose outside the target range with disordered eating motivations	Opposite of active Relates to health behavior
Insulin inflation	Active and passive addition of insulin to a prescribed dose with disordered eating motivations, such as deliberate administration of a higher dose than prescribed, or deliberate failure to proportionately reduce a dose in preparation for exercise or other activity that results in a lowering of blood glucose.	Avoids Mental Disorder connotations not related to disordered eating (Factitious Disorder, Borderline Personality Disorder).
Insulin restriction	Active and passive removal of insulin from a prescribed dose with disordered eating motivations, such as deliberate administration of a lower dose than prescribed, or failure to administer insulin to correct a high glucose not induced intentionally	Avoids connotations of Bulimia Nervosa with “Diabulimia.” Restriction can be defined latterly as partial or total, where insulin omission implies total and active failure to administer a dose.

IRDEB, a term not used in previous studies, is proposed to capture insulin manipulation where there is a food or weight‐related motivation. IRDEB provides an umbrella term that encompasses the manipulation of insulin of all insulin activity profiles: active and passive, positive and negative insulin manipulation; and all disordered eating motivations relating to these behaviors. This term also excludes adherence‐based motivations and avoids connotations with clinical eating disorders.

The use of “active” and “passive” categorization of IRDEB arises from the categories of insulin manipulation identified from the behaviors of interest charted across all sources. This framing also avoids the blame connotations of the term “intentional” used in previous literature. This approach, derived from Health Psychology principles, refers to the difference between actively inducing hypo‐ or hyperglycemia and passively allowing prior actions not originally intended to induce hypo‐ or hyperglycemia to induce these states for eating‐ or weight‐related motivations. An example of *actively* engaging in insulin restriction to induce hyperglycemia would be intentionally reducing a dose of insulin relative to the carbohydrate content of a meal or snack. Comparatively, *passive* insulin restriction would be noticing an unintentional trend towards hyperglycemia, perhaps as a result of miscalculated bolus or the impact of a non‐carbohydrate element of a drink or meal (such as fat or alcohol) and not intervening with a correction dose to remain in or return to the target glucose range.

*Insulin restriction* is the term proposed to encompass removal of insulin from a prescribed dose with disordered eating motivations. This was chosen as the third‐most frequently used term in the literature to minimize impact of changing terms. Insulin restriction is suggested over insulin omission, as “omission” implies the complete removal of a regime or dose, where “restriction” can be quantified as partial or total, moving away from the “all or nothing” mentality that has the potential to disregard individuals engaging in IRDEB on a functional level. “Diabulimia” was disregarded as a term despite the community identifying with this term in the 2010s (Allan 2015) due to the misleading associations with Bulimia Nervosa highlighted (e.g., see Wisting and Snoek 2020) and movement away from the term in professional settings (Partridge et al. 2020).

*Insulin inflation* is the term proposed to encompass addition of insulin to a prescribed dose with disordered eating motivations. Despite “insulin overdose” being the most common phrase used, this term invites connotations and literature surrounding behaviors relating to self‐harm and suicide and Borderline Personality Disorder, as well as perpetuating a similar “all or nothing” mentality as described above with regards to omission and restriction. Similarly, “Factitious Hypoglycemia” was disregarded due to its invitation of connotations with Factitious Disorder (née Munchausen's Syndrome).

## 4 Discussion

This paper provides the first comprehensive review of terminology used to refer to disordered eating behaviors involving insulin manipulation in T1D. Given the potential harm caused by stigma surrounding disordered eating and diabetes mismanagement and related physical consequences, it is important to consolidate terms in the area to harmonize existing evidence and promote communication and research. Therefore, this brief report aimed to summarize terminology used in research investigating disordered eating behavior involving insulin manipulation and developed terms to be used going forward.

Across the literature, there was little reference to the motivation underlying insulin manipulation in the phrases used to identify behaviors of interest. This invites uncertainty about participants' interpretation of measures used to evaluate IRDEB, in the lack of distinction from non‐adherence unrelated to disordered eating, such as avoidance of the pain of injections and motivations relating to self‐harm and suicide. To reflect this, the umbrella term “insulin‐related disordered eating behaviour” is proposed to include insulin‐related behaviors relating to disordered eating motivation (i.e., basal or bolus manipulation, complete or partial restriction, inflation to control the urge to binge eat or consume ‘additional’ food to that which dietary rules prescribe, or failure to correct high or low blood sugars to achieve goals relating to disordered eating).

This term transcends strict eating disorder diagnostic labels whilst still being related to eating behavior, as the behaviors are not mutually exclusive or inclusive of clinical diagnosis with current DSM‐5 (American Psychiatric Association 2022) or ICD‐11 guidelines (World Health Organisation 2022). In addition to capturing the range of behaviors and related impairment, rather than limiting the weight loss motivations that are shown to be less prevalent in IRDEB (Pigott et al. 2024), this reflects the desire of individuals engaging in insulin restriction to be separated from other clinical eating disorder populations (Goddard and Oxlad 2023). To communicate taking a lower insulin dose than prescribed, the term “insulin restriction” is recommended instead of “insulin omission” given that insulin *restriction* describes the removal of insulin and can encompass partial or complete restriction, whereas “insulin omission” implies the total exclusion of a dose or regime. The use of “restriction” over “omission” ameliorates the “all or nothing” rhetoric present in some literature (Martin et al. 2023), allowing space for individuals to be “functioning” with these behaviors rather than completely omitting all insulin and diabetes regimen and raising red flags with Diabetic Ketoacidosis (DKA) admissions and similar (Eilander et al. 2017; Partridge et al. 2020). For taking more insulin than the prescribed dose, the term “insulin inflation” is suggested to distinguish the disordered eating behavior from the nuances of self‐harm and suicidal ideation relating to “overdose.” A key consideration moving forward is to consider the preferences of individuals engaging in IRDEB when advocating for and labelling official diagnostic criteria and care pathways, given literature that suggests a strong identity with current “diabulimia” and “diabulimic” labels (Allan 2015; Goddard and Oxlad 2023).

The use of a scoping review methodology is a strength of this research. Methods of searching, inclusion of sources, and data charting were systematic, rigorous, and pre‐registered, thus minimizing potential bias in the gathering and analysis of evidence relevant to the review of current and development of new terminology. A limitation is the inability to obtain some of the full texts of sources that made it to the full text screening stage of the review process.

Based on the findings of this comprehensive review, IRDEB is the term proposed for future use as it encompasses facets of active and passive insulin restriction and insulin inflation, and separates these behaviors from eating behaviors not related to the manipulation of insulin as well as insulin manipulation not related to disordered eating. It is hoped that consistent use of this term will consolidate and harmonize knowledge and communication across research and practice, and will help clarify the field across all modalities accessed by patients, families, and clinicians.

## Author Contributions

**Amy Shelford:** conceptualization, formal analysis, investigation, writing – original draft, writing – review and editing, project administration, visualization, data curation. **Paul E. Jenkins:** writing – review and editing, supervision. **Kate Harvey:** conceptualization, writing – review and editing, supervision.

## Conflicts of Interest

The authors declare no conflicts of interest.

## Supporting information


**Data S1:** eat24548‐sup‐0001‐Supinfo1.docx.

## Data Availability

The data that support the findings of this study are openly available in Open Science Framework at https://osf.io/yu3tk/files/osfstorage.
