# Supplementary material for: Insulin‐Related Disordered Eating Behavior: Clarifying Terminology
Source: Int J Eat Disord. 2025 Sep 16;58(12):2437–43. doi: 10.1002/eat.24548 (PMC12703216; doi:10.1002/eat.24548)
Supplement: Supplementary file 1 — Data S1: eat24548‐sup‐0001‐Supinfo1.docx. [file EAT-58-2437-s001.docx]

**Ovid: PsycINFO**

|  | Title, abstract, heading word, table of contents, key concepts, original title, tests & measures, mesh word | (diabetes and (“type 1” or “type I”)) OR “insulin dependent diabetes” OR “juvenile diabetes” |
| --- | --- | --- |
| AND | Title, abstract, heading word, table of contents, key concepts, original title, tests & measures, mesh word | "insulin omission" or "insulin restriction" or "insulin manipulation" or "insulin abuse" or "insulin overdose" or (insulin and “disordered eating behavio*”) or diabul* or T1DE |
| NOT | Title, abstract, heading word, table of contents, key concepts, original title, tests & measures, mesh word | suicid* |

**PubMed**

|  | All fields | (diabetes AND ("type 1" OR "type I")) OR "insulin dependent diabetes" OR "juvenile diabetes") |
| --- | --- | --- |
| AND | All fields | ("insulin omission" OR "insulin restriction" OR "insulin manipulation" OR "insulin overdose" OR "insulin abuse" OR (insulin AND "disordered eating behavio*") OR diabul* OR T1DE) |
| NOT | All fields | Suicid* |

**Scopus**

|  | Title Abstract& Keywords | ( diabetes AND ( "type 1" OR "type I" ) ) OR "insulin dependent diabetes" OR "juvenile diabetes" |
| --- | --- | --- |
| AND | Title Abstract& Keywords | “insulin omission” OR "insulin restriction" OR "insulin manipulation" OR "insulin overdose" OR "insulin abuse" OR (insulin AND “disordered eating behavio*”) OR diabul* OR t1de |
| AND NOT | Title Abstract& Keywords | suicid* |

**Web of Science**

|  | Topic | ( diabetes AND ( "type 1" OR "type I" ) ) OR "insulin dependent diabetes" OR "juvenile diabetes" |
| --- | --- | --- |
| AND | Topic | “insulin omission” OR "insulin restriction" OR "insulin manipulation" OR "insulin overdose" OR "insulin abuse" OR (insulin AND “disordered eating behavio*”) OR diabul* OR t1de |
| NOT | Topic | suicid* |

**ProQuest:** Ebook Central; IBSS; Periodicals Archive Online; Academic Complete; Australia And New Zealand Database; Biological Science Database; Consumer Health Database; Continental Europe Database; East & South Asia Database; East Europe, Central Europe Database; Health & Medical Collection; Healthcare Administration Database; India Database; Latin America & Iberia Database; Middle East & Africa Database; Nursing And Allied Health Database; Psychology Database; Public Health Database; Publicly Available Content Database; Research Library; Science Database; Social Science Database; Turkey Database; UK And Ireland Database; Proquest Dissertations & Theses Global

|  | Summary | diabetes and ("type 1" or "type I")) or "insulin dependent diabetes" or "juvenile diabetes") |
| --- | --- | --- |
| AND | Summary | "insulin omission" OR "insulin restriction" OR "insulin manipulation" OR "insulin overdose" OR "insulin abuse" OR (insulin AND "disordered eating behavio*") OR diabul* OR T1DE |
| NOT | Summary | Suicid* |

**Open Science Framework**

"type 1 diabetes" AND insulin

**Cochrane Library**

"type 1 diabetes" and insulin

**PROSPERO**

"insulin omission" OR "insulin restriction" OR "insulin manipulation" OR "insulin abuse" OR "insulin overdose" OR diabul* OR T1de

**Diabulimia Helpline**
